# Human TTBK1, TTBK2 and MARK1 kinase toxicity in *Drosophila melanogaster* is exacerbated by co-expression of human Tau

**DOI:** 10.1242/bio.022749

**Published:** 2017-07-15

**Authors:** Josefin Fernius, Annika Starkenberg, Malgorzata Pokrzywa, Stefan Thor

**Affiliations:** Department of Clinical and Experimental Medicine, Linkoping University, Linkoping SE-581 85, Sweden

**Keywords:** Tauopathy, Neuronal toxicity, *Drosophila*, Tau-kinases

## Abstract

Tau protein is involved in numerous human neurodegenerative diseases, and Tau hyper-phosphorylation has been linked to Tau aggregation and toxicity. Previous studies have addressed toxicity and phospho-biology of human Tau (hTau) in *Drosophila melanogaster*. However, hTau transgenes have most often been randomly inserted in the genome, thus making it difficult to compare between different hTau isoforms and phospho-mutants. In addition, many studies have expressed hTau also in mitotic cells, causing non-physiological toxic effects. Here, we overcome these confounds by integrating *UAS-hTau* isoform transgenes into specific genomic loci, and express hTau post-mitotically in the *Drosophila* nervous system. Lifespan and locomotor analyses show that all six of the hTau isoforms elicit similar toxicity in flies, although hTau^2N3R^ showed somewhat elevated toxicity. To determine if Tau phosphorylation is responsible for toxicity, we analyzed the effects of co-expressing hTau isoforms together with Tau-kinases, focusing on TTBK1, TTBK2 and MARK1. We observed toxicity when expressing each of the three kinases alone, or in combination. Kinase toxicity was enhanced by hTau co-expression, with strongest co-toxicity for TTBK1. Mutagenesis and phosphorylation analysis indicates that hTau-MARK1 combinatorial toxicity may be due to direct phosphorylation of hTau, while hTau-TTBK1/2 combinatorial toxicity may result from independent toxicity mechanisms.

## Abstract (precis)

**Summary:** Tau hyper-phosphorylation has been linked to toxicity, but the Tau isoforms, kinases and residues remain unclear. Using the *Drosophila* model, we find evidence for involvement of TTBK and MARK kinases.

## INTRODUCTION

Tau is a neuronal, microtubule-associated protein that plays a key role during microtubule assembly and stabilization. Tau is also heavily implicated in human disease, in particular in Alzheimer's disease (AD) where intracellular neurofibrillary tangles (NFTs) of hyper-phosphorylated human Tau (hTau) is one of the two major histopathological hallmarks found in patients with the disease (Braak and Braak, 1991). Additional tauopathies have been identified, characterized by intracellular aggregates of hyper-phosphorylated hTau as well as mutations in hTau, e.g. fronto-temporal dementia with parkinsonism-17 (FTDP-17), Pick's disease and corticobasal degeneration (CBD) (Coppola et al., 2012; Kouri et al., 2014). Although the pathophysiology of hTau in these neurodegenerative diseases has been intensively studied over recent years, its contribution to disease initiation and progression remains unclear.

**Fig. 1.**
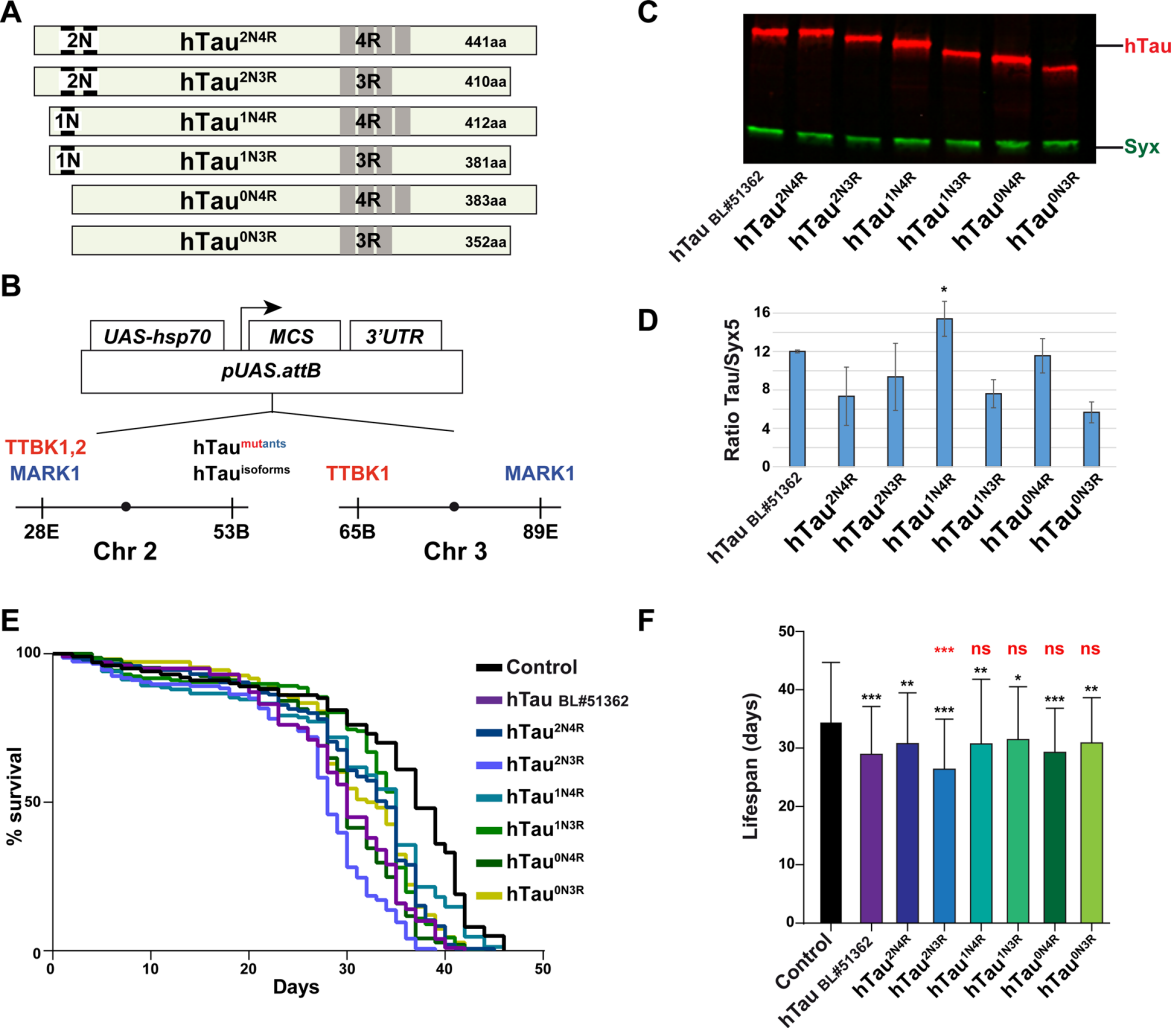
**Expression of human Tau isoforms in post-mitotic neurons results in reduced lifespan in *Drosophila.*** (A) Schematic diagram of the six main hTau isoforms, which vary in the N-terminal domains and the R-repeats. (B) Constructs were inserted in the *pUAS.attB* vector, and landed at the indicated *attP* landing sites. (C) Western blot analysis of hTau protein levels in fly head extracts, using Odyssey CLX Licor system. Syntaxin (Syx) was used as a loading control. (D) Quantification of protein expression using Licor Image Studio software, showing average signal ratio of hTau/Syx (three independent experiments, 20 adult heads each; asterisks depict pair-wise comparison with hTau^2N4R^; * *P*≤0.05; Student's two-tailed *t*-test; mean±s.d.). (E,F) Lifespan assay measuring the toxicity of the six common hTau isoforms, landed in the same genomic location, driven by the post-mitotic, pan-neuronal *n-Syb-Gal4* driver. Results are depicted as Kaplan–Meier survival curves (E) (Kaplan and Meier, 1958) and bar graph (F). Control flies (*n-Syb-Gal4* crossed to the *attP* landing site strain, BL#9750) were compared to a previously published human Tau^2N4R^ construct (*UAS-hTau* BL#51362), and all six isoforms of *UAS-hTau* landed on chromosome 2 (53B). Three independent experiments were performed, which gave similar results, and hence the graphs show the average lifespan of the combination of the three experiments (≥100 flies). Error bars in (F) indicate mean±s.d.; black asterisks depict pair-wise comparison with control, and red asterisks depict pair-wise comparison with hTau^2N4R^ (*P≤0.05, **P≤0.01, ***P≤0.001; Student's two-tailed *t*-test); ns, not significant.

In the adult human brain, there are six main hTau isoforms, ranging from 352 to 441 amino acids, which are differentially expressed during development (Goedert et al., 1989; Takemura et al., 1991; Takuma et al., 2003). These isoforms derive from alternative mRNA splicing of the hTau gene (van Slegtenhorst et al., 2000), and differ from each other in the absence or presence of two N-terminal domains (N1, N2), and three or four carboxy-terminal microtubule binding domains (R1-R4) (Fig. 1A). These isoforms may have specific roles during development, for example with regards to axonal generation and their role in microtubule cytoskeleton stability (van Slegtenhorst et al., 2000). However, it has also become clear that specific isoforms are either more abundant or absent from brains of patients with various forms of dementia (Andreadis, 2012; Niblock and Gallo, 2012). Tau has an unusually large proportion of phosphorylation sites; the 2N4R isoform has a total of 441 amino acids with 85 serines, threonines and tyrosines, 45 of which have been observed to be phosphorylated (Hanger et al., 2009). Tau phosphorylation is also regulated developmentally, and during early fetal development Tau is highly phosphorylated and modified at many sites, broadly similar to the phosphorylation status of Tau in AD (van Slegtenhorst et al., 2000). Interestingly, although highly phosphorylated, Tau is functional in the fetus and does not form NFTs as it does in AD.

Several protein kinases and phosphatases regulate Tau phosphorylation state (Wang and Mandelkow, 2016). Genetic screens in *Drosophila* and *Caenorhabditis elegans* have identified several kinases as suppressors/enhancers of Tau toxicity (Shulman and Feany, 2003; Kraemer et al., 2006; Ambegaokar and Jackson, 2011). Among these are the microtubule affinity regulating kinase (MARK) and Tau tubulin binding kinase1 and 2 (TTBK1, TTBK2)*.*

*Drosophila* has been extensively used to study Tau biology due to its sophisticated genetic tools, short lifespan, relative low cost, and the powerful *Gal4/UAS* transgenic expression system (Gistelinck et al., 2012; Fernandez-Funez et al., 2015; Sun and Chen, 2015). However, previous studies mostly used *P* element *UAS* transgenes, which randomly insert into the genome and hence are susceptible to position effects and copy number complications. This problem makes it difficult to compare numbers of Tau isoforms and conduct controlled structure-function studies. Moreover, many studies have relied on expressing hTau both in progenitor and post-mitotic cells, using the eye *Gal4* driver *GMR-Gal4*, expressed throughout eye disc development, or the nervous system driver *elav-Gal4*, which also expresses in actively dividing neural progenitor cells (Freeman, 1996; Berger et al., 2007). Because Tau is a microtubule-binding protein, it will likely interfere with mitosis in cycling cells, a notion that was recently confirmed by detailed studies in the wing disc (Bouge and Parmentier, 2016).

To circumvent these problems we have (1) used *attP UAS* ‘landing sites’ to avoid position and copy number confounds (Bischof et al., 2007), and (2) used post-mitotic neuronal *Gal4* drivers, either expressed throughout life or specifically induced in adults. Combined, this enables precise comparative structure-function analysis of hTau isoforms and phospho-sites, and addresses the effects of various kinases on toxicity. Using these improvements, we addressed hTau isoform toxicity, and found similar toxicity for all of the six main hTau isoforms, although hTau^2N3R^ showed somewhat elevated toxicity. We furthermore analyzed the toxic effects of co-expressing hTau with three Tau-directed kinases: TTBK1, TTBK2, and MARK1. We observed combinatorial hTau-kinase toxicity, as judged by reduced lifespan and locomotor activity, in particular when co-expressing the TTBK1 kinase along with hTau. Focusing of TTBK1 and hTau, we find similar combinatorial toxicity for all six hTau isoforms. Phospho-residue mutagenesis of hTau, as well as phosphorylation analysis, indicates that in the case of MARK1, combinatorial toxicity may be due to kinase acting directly upon hTau. In contrast, the enhanced toxicity with TTBK1, -2, does not appear to be related to altered hTau phosphorylation.

## RESULTS

### Expression of the six main human Tau isoforms in post-mitotic neurons in *Drosophila* results in moderate toxicity

We generated DNA constructs for the six main hTau isoforms (Fig. 1A) by gene synthesis. Open reading frames were codon-optimized for *Drosophila* expression, and a ‘Cavener start-ATG’ was added to the 5′ (Cavener and Ray, 1991) (see Supplementary Materials). Constructs were inserted into the *pUAS.attB* landing site transgenic vector (Bischof et al., 2007) and all six isoform transgenes were landed at the same site on chromosome 2 (53B) (Fig. 1B). To enable robust, post-mitotic expression in most neurons in the nervous system, we used the previously described *n-Syb-Gal4* driver; a driver where *Gal4* expression is under control of the neuronal *Synaptobrevin* promoter (Jonson et al., 2015). Combined with landing site transgenesis, this enables quantitative comparison of toxicity of the six different hTau isoforms in post-mitotic neurons.

Immunoblot analysis indicated robust expression of all six isoforms of hTau, with similar expression levels, although hTau^1N4R^ did show a significantly increased protein expression when compared to the hTau^2N4R^ (Fig. 1C,D). Lifespan experiments revealed that all six hTau isoforms showed significant reduction of lifespan when compared to control flies (*n-Syb-Gal4* crossed to the *attP* landing site strain BL#9750), with evidence for a somewhat elevated toxicity for hTau^2N3R^ when compared to the hTau^2N4R^ (Fig. 1E,F). We also analyzed a previously published human *UAS-Tau* transgene for comparison (Chatterjee et al., 2009). This transgene displayed expression levels and a reduction in lifespan that was generally comparable to our novel transgenes (Fig. 1C-F).

### Expression of the TTBK1, TTBK2 and MARK1 hTau-directed kinases reveals strong toxicity for TTBK1 and TTBK2

**Fig. 2.**
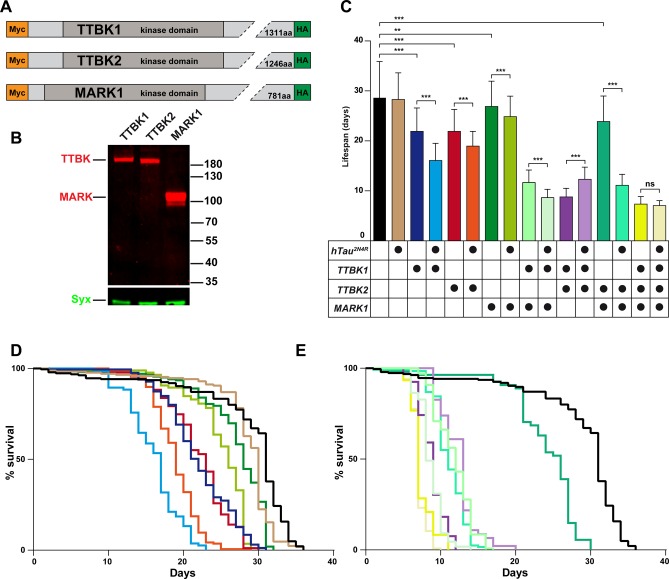
**MARK1 and TTBK1, TTBK2 kinases reduce lifespan, and the effects are increased by co-expression with hTau.** (A) Human MARK and TTBK kinase constructs; a c-Myc and HA antibody epitope tag was fused to the N- or C-terminus, for detection by western blot. (B) Western blot analysis of fly head extracts expressing human TTBK or MARK kinases. Kinases were detected with anti-HA antibody, and Syx was used as loading control. There is robust protein expression of all three kinases. TTBK1 and TTBK2 migrated substantially slower than their molecular weight. (C-E) Lifespan analysis of flies expressing kinases with or without co-expression of hTau^2N4R^, using the inducible *n-Syb-Gal4;Gal80[ts]* driver. Results are depicted as Kaplan–Meier survival curves (D,E) (Kaplan and Meier, 1958) and bar graph (C). Flies were crossed and reared at 20°C until they hatched, then transferred to 29°C. Using this inducible driver, there was no significant reduction in lifespan of hTau^2N4R^ when compared to control flies (*n-Syb-Gal4;Gal80[ts]* crossed to the *attP* landing site strain BL#9750). However, all other flies showed significantly reduced lifespan when compared to control flies, including flies expressing each of the three kinases alone. There was combinatorial toxicity when co-expressing hTau^2N4R^ with hTau-directed kinases TTBK1, TTBK2 or MARK1. The graphs show the average lifespan from a combination of 3-5 independent experiments (≥46 flies). Error bars in (C) indicate mean±s.d. (*P≤0.05, **P≤0.01, ***P≤0.001; ANOVA multiple comparison Tukey's test).

The low toxicity observed upon post-mitotic expression of hTau in the *Drosophila* CNS facilitated the systematic testing of combinatorial toxicity of hTau when co-expressed with hTau-directed kinases; TTBK1, TTBK2 and MARK1 kinases were identified in genetic screens (Shulman and Feany, 2003; Kraemer et al., 2006; Ambegaokar and Jackson, 2011). We generated *UAS* attB transgenes for these kinases (Fig. 2A), codon-optimized for expression in *Drosophila*, and tagged at N- and C-terminals with Myc and HA epitope tags, respectively (Fig. 2A; see Supplementary Materials). All three kinase transgenes were integrated into the same site on chromosome 2 (28E) (Fig. 1B). To assess the combinatorial contribution of multiple kinases to hTau-related toxicity, TTBK1 and MARK1 kinase transgenes were also integrated at site 65B or 89E, respectively, on chromosome 3 (Fig. 1B).

First, we analyzed protein expression using western blot of fly head protein extracts, followed by detection using the epitope antibodies, and noted that all three kinases were readily detectable (Fig. 2B). Next, we addressed the toxicity of each kinase when expressed alone, without hTau, by crossing them to the *n-Syb-Gal4* driver. Expression of TTBK1 kinase was larval lethal, with no adult flies emerging, and expression of TTBK2 kinase also elicited severe toxic effects with very few adults emerging. These results indicate that broad post-mitotic neuronal expression of TTBK1 and TTBK2 kinases is toxic to the fly, even without co-expression of hTau.

### Inducible expression of TTBK1, TTBK2 and MARK1 kinases along with hTau^2N4R^ results in combinatorial toxicity

Because of complete lethality of TTBK1 and partial lethality of TTBK2 using the *n-Syb-Gal4* driver, we generated an inducible driver to enable development into adulthood before inducing expression. To achieve this, we used *Gal80[ts]*, a temperature-sensitive *Gal80* allele that represses Gal4 protein activity at 20°C, but allows Gal4 activity at 29°C (McGuire et al., 2003). We generated flies carrying two copies of *n-Syb-Gal4* and two copies *tubP-Gal80[ts]* transgenes, for robust Gal4 and Gal80[ts] activities. These flies were crossed to *UAS-eGFP*, and the resulting progeny did not express eGFP if maintained at 18°C, but eGFP was strongly induced in all neurons after shifting flies to 29°C as adults (Fig. S1). Crossing this driver to the *UAS-TTBK1* and *UAS-TTBK2* kinase transgenes allowed adults to emerge, enabling comparison of toxicity with *UAS-hTau* alone. Because kinases are known to activate each other by priming phosphorylation, we generated flies expressing one, two or three kinases, with or without hTau to analyze combinatorial toxic effects on hTau.

Focusing first on the kinases, using the inducible *n-Syb-Gal4;Gal80[ts]* driver we noted reduced lifespan as a result of expressing the TTBK1, TTBK2 or MARK1 kinases, when compared to control (*n-Syb-Gal4;Gal80[ts]* crossed to the *attP* landing site strain BL#9750) (Fig. 2C-E; Table S1). Because kinases have been found to prime each other by phosphorylating neighboring residues in proteins (Cesaro and Pinna, 2015), we generated flies carrying multiple *UAS* kinase transgenes. Flies expressing multiple kinases showed enhanced toxicity compared to fly strains expressing a single kinase. This was true both for TTBK1-TTBK2 co-expression, as well as for TTBK1-MARK1 co-expression. Surprisingly, TTBK2-MARK1 co-expression did however not show enhanced toxicity (Fig. 2C-E; Table S1).

Turning next to hTau-kinase co-expression, we first noted that hTau^2N4R^ expression alone, using adult-induced Gal4 activity, caused no significant reduction in lifespan. However, adult-induced co-expression of the TTBK1, TTBK2 or MARK1 kinase with hTau^2N4R^ showed a significant combinatorial toxicity (Fig. 2C-E; Table S1). This combinatorial effect was strongest for TTBK1. Co-expression of both TTBK1 and MARK1 kinases with hTau^2N4R^ resulted in further increased toxicity. Similarly, TTBK2-MARK1 co-expression, which was weakly toxic on its own, showed severe toxicity together with hTau^2N4R^ (Fig. 2C-E; Table S1). In contrast, the strong toxicity of TTBK1, TTBK2 co-expression was somewhat alleviated by co-expression of hTau^2N4R^. Expression of the three kinases together was strongly toxic, and was not further enhanced by co-expression of hTau^2N4R^. These results reveal that co-expression of kinases results in combinatorial toxicity, and that co-expression of kinases together with hTau^2N4R^ results in enhanced combinatorial toxicity.

### All six hTau isoforms are combinatorially toxic with TTBK1

**Fig. 3.**
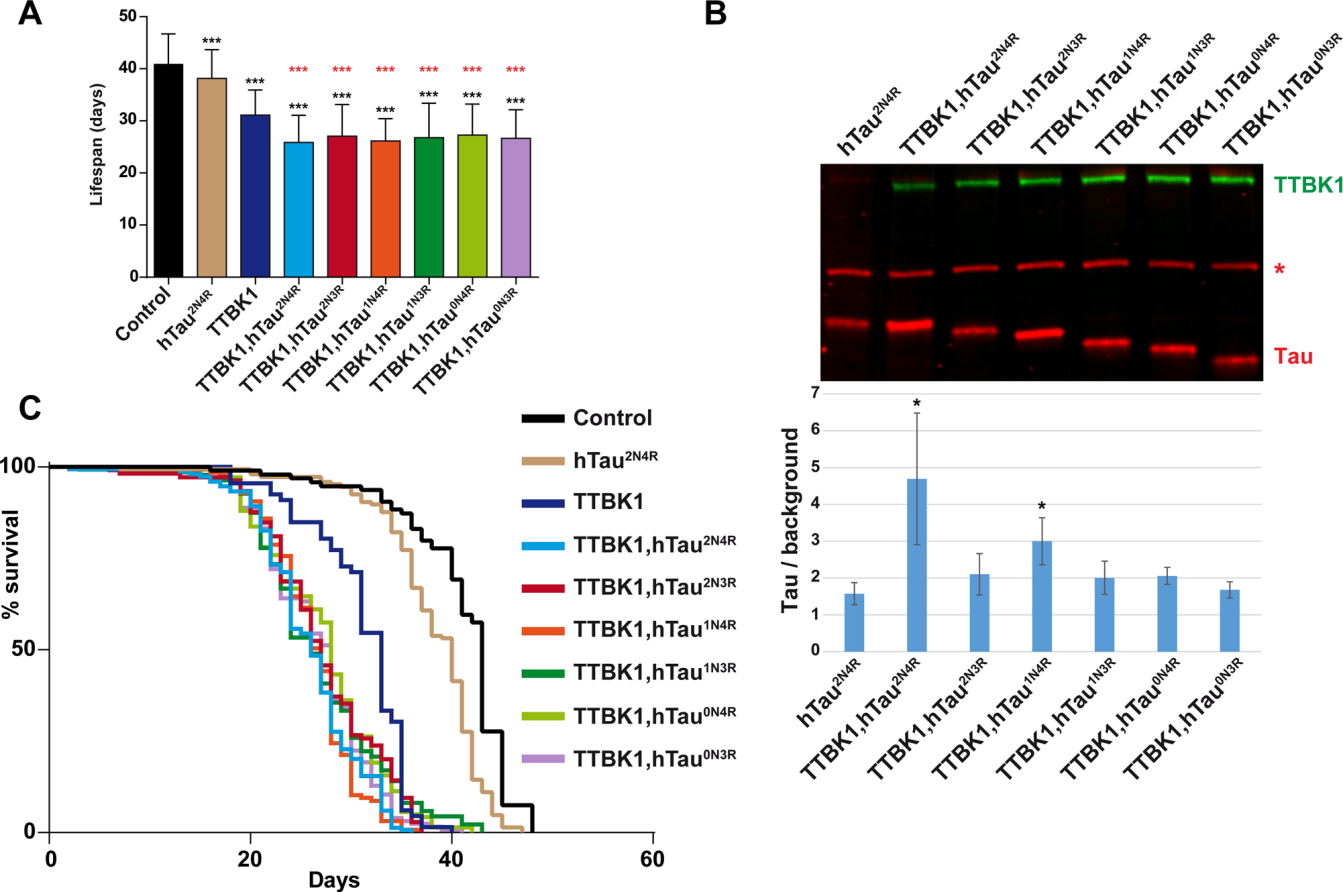
**TTBK1 toxicity is enhanced by all six hTau isoforms.** (A,B) Lifespan and western blot analysis of flies expressing TTBK1 with hTau isoforms, using the inducible *n-Syb-Gal4;Gal80[ts]* driver. Results are depicted as Kaplan–Meier survival curves (C) (Kaplan and Meier, 1958) and bar graph (A). (A,C) All six hTau isoforms showed significantly shortened lifespan when co-expressed with TTBK1, when compared to control flies (*n-Syb-Gal4;Gal80[ts]* crossed to the *attP* landing site strain BL9750). Error bars in (A) indicate mean±s.d.; black asterisks depict pair-wise comparison with control, and red asterisks depict pair-wise comparison with TTBK1 (***P≤0.001; Student's two-tailed *t*-test; *n*=3 independent experiments; ≥70 flies). (B) Western blot, using Licor, of fly head extracts after 5 days expression of the transgenes at 29°C. TTBK1 kinase was detected using anti-HA antibody, hTau was detected using a rabbit anti-hTau antibody (asterisk denotes antibody background reactivity to an endogenous *Drosophila* protein). We observed increased hTau levels in the hTau^2N4R^ and hTau^1N4R^ isoforms when co-expressed with TTBK1. Bar graph in B represents the average value of three independent gels, showing the value of the ratio of Tau/ background. Error bars indicate mean±s.d. and black asterisks depict pair-wise comparison with hTau^2N4R^.

The above experiments were conducted using the 2N4R isoform of hTau. Next, we tested whether the combinatorial hTau-kinase toxicity was similar for all six different hTau isoforms, focusing on the TTBK1 kinase, due to the strong effects of TTBK1 on lifespan. To this end, the other five isoforms, landed in the same genomic location as 2N4R, were also combined with TTBK1. We noted similar combinatorial effects on lifespan between TTBK1 and all six hTau isoforms when compared to control (*n-Syb-Gal4;Gal80[ts]* crossed to the *attP* landing site strain BL#9750) (Fig. 3A,C). Analyzing hTau protein levels we noted significantly elevated hTau^2N4R^ and hTau^1N4R^ protein levels when co-expressed with TTBK1 (Fig. 3B).

### Co-expression of TTBK1, TTBK2 or MARK1 kinase with hTau^2N4R^ in motor neurons resulted in combinatorial locomotor defects

**Fig. 4.**
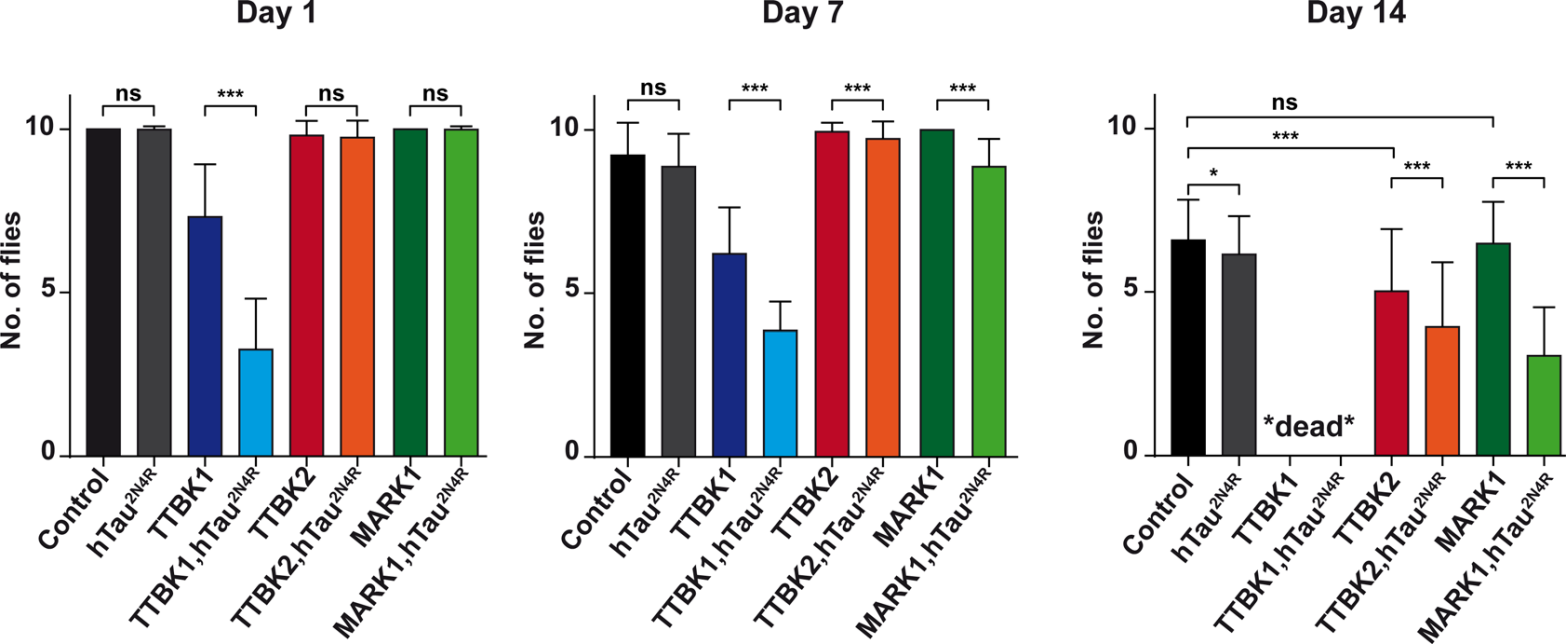
**Combinatorial geotaxis effects between hTau and TTBK/MARK kinases.** Negative geotaxis assay shows combinatorial effects from co-expressing hTau^2N4R^ with TTBK1, TTBK2 or MARK1 kinase, expressed using the *OK371-Gal4* driver. Flies were assessed for climbing ability at Day 1, 7 and 14. Ten empty vials with ten flies per vial were counted ten times each, and the average number of flies per vial that climbed up to the 5 cm mark within 30 s is plotted in the graphs (error bars indicate mean±s.d.; *P≤0.05, **P≤0.01, ***P≤0.001; Student's two-tailed *t*-test; *n*=3 independent experiments; ≥100 flies). Expression of each kinase alone revealed effects of TTBK1 at Day 1 and Day 7, and for TTBK2 at Day 14, when compared to control flies (*OK371-Gal4* crossed to the *attP* landing site strain BL#9750). At Day 14, TTBK1 flies had died. MARK1 expression alone showed no toxicity. Co-expression revealed that on Day 1 there was significant difference between flies expressing TTBK1 when compared to TTBK1-hTau^2N4R^ flies, and this effect persisted into Day 7; by Day 14 these genotypes had died. On Day 7 there was significant difference between flies expressing MARK1 when compared to MARK1-hTau^2N4R^, as well as between TTBK2 and TTBK2-hTau^2N4R^. These effects persisted into Day 14. Expression of hTau^2N4R^ alone showed significant only at Day 14. ns, not significant.

To further analyze combinatorial hTau-kinase toxicity, we complemented the lifespan assays with a motor output behavioral assay. For these studies we used *OK371-Gal4*, which drives expression in most/all fly motor neurons (Mahr and Aberle, 2006). We used a negative geotaxis assay, where the flies' ability to climb up the side of a vial is assessed, at days 1, 7 and 14 after emerging as adults. Briefly, the percentage of flies climbing up to a 5 cm mark on the vial within 30 s was quantified (see Materials and Methods). Flies expressing hTau^2N4R^ alone did not show any significant effect upon climbing ability when compared to control at Day 1 and 7, but a minor effect at Day 14 (Fig. 4). In contrast, flies expressing TTBK1 alone displayed significantly reduced climbing ability when compared to control (*OK371-Gal4* crossed to the *attP* landing site strain BL#9750), apparent already at Day 1 and more pronounced at Day 7. By Day 14, all TTBK1 flies had died (Fig. 4). Expression of TTBK2 alone showed milder but significant effects, apparent only at Day 14, while MARK1 alone showed no effects (Fig. 4).

Next, we turned to addressing possible combinatorial effects. We found that the climbing ability of flies expressing TTBK1, TTBK2 or MARK1 kinases were all significantly reduced by the co-expression of hTau^2N4R^ (Fig. 4). These effects were apparent already at Day 1 for TTBK1-hTau^2N4R^, and at Days 7 and 14 for TTBK2-hTau^2N4R^ and MARK1-hTau^2N4R^ (Fig. 4). We attempted to test additional combinations of these three kinases with or without hTau^2N4R^ in the climbing assay, but all such *UAS* combinations were larval lethal using the *OK371-Gal4* driver, and adult flies never emerged.

We conclude that expression of hTau^2N4R^ in motor neurons, using the *OK371-Gal4* driver, has no effect upon locomotor activity, i.e. climbing ability. Single expression of TTBK1 or TTBK2 kinases affected geotaxis, while MARK1 had no effect. The TTBK1, TTBK2, effects were worsened by co-expression of hTau^2N4R^. MARK1 also had effects when co-expressed with hTau. Combinations of two or three kinases, with and without hTau, were larval lethal using the *OK371-Gal4* driver.

### Co-expression of hTau with kinases increases phosphorylation

The combinatorial toxicity observed when we co-expressed hTau with kinases in this system may be caused by increased phosphorylation of hTau by the kinases. To investigate this we used pan-hTau and phospho-specific hTau primary antibodies, and fluorescent secondary antibodies to determine relative levels of specific phospho-hTau epitopes, when compared to pan-hTau levels, using adult fly head extracts. Due to the lethality of TTBK1, and partial lethality for TTBK2, we again used the inducible *n-Syb-Gal4;Gal80[ts]* driver to obtain adult heads.

**Fig. 5.**
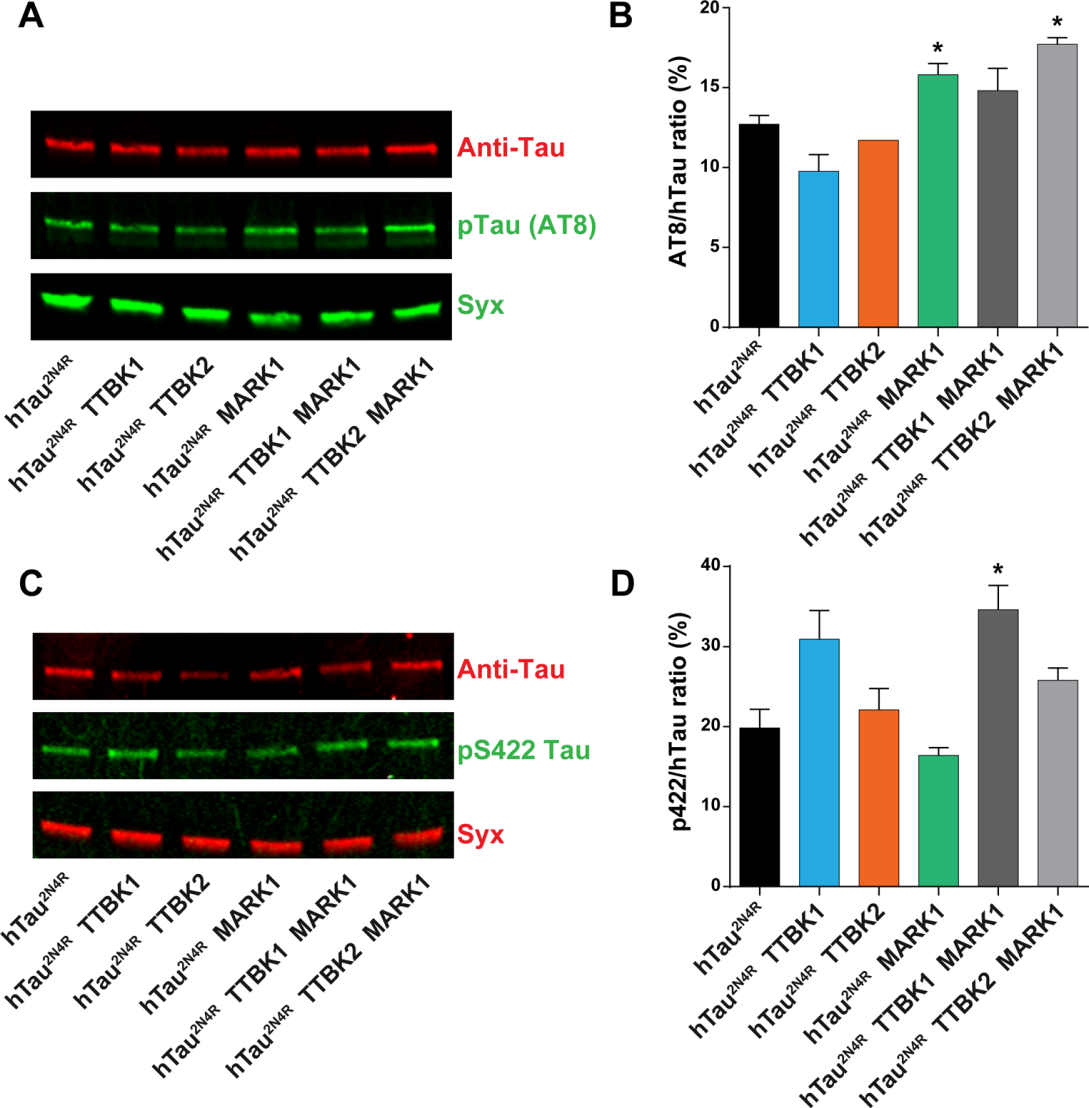
**Phospho-analysis of flies expressing hTau^2N4R^ and Tau kinases.** (A,C) Western blot, using Licor, of fly head extracts after 5 days expression of the transgenes at 29°C (two independent experiments, 20 adult heads each). Transgene expression was induced by incubating the flies at 29°C for five days using the *n-Syb-Gal4;Gal80[ts]* driver. (A) Total hTau^2N4R^ was detected using a rabbit hTau antibody and phospho-S202 and S205 were detected using mouse monoclonal AT8 antibody. Syx was used as a loading control. (B) Quantification of AT8/total hTau from two similar experiments in A, using Licor software Studio Image. We observed an increase in the AT8/total hTau ratio in flies expressing MARK1-hTau^2N4R^ and TTBK2-hTau^2N4R^-MARK1, when compared to hTau^2N4R^ alone (error bars indicate mean±s.d.; *P≤0.05; Student's two-tailed *t*-test). (C) Total hTau was detected using a mouse hTau5 antibody and phospho-S422 was detected using rabbit polyclonal pS422 antibody. Syx was used as loading control. (D) Quantifications of pS422/total hTau from two similar experiments in C, using Licor software Studio Image. We observed an increase in the AT8/total hTau ratio in flies expressing TTBK1-hTau^2N4R^-MARK1, when compared to hTau^2N4R^ alone (error bars indicate mean±s.d.; *P≤0.05; Student's two-tailed *t*-test).

The AT8 monoclonal antibody detects phosphorylated S202 and T205, epitopes known to be phosphorylated by TTBK1 and TTBK2. We observed that hTau^2N4R^ extracts stained for AT8, even without kinase co-expression, and hence hTau^2N4R^ is already phosphorylated at these sites by endogenous *Drosophila* kinases (Fig. 5A). Surprisingly, we found that the levels of AT8 in hTau^2N4R^-TTBK1 or hTau^2N4R^-TTBK2 co-expression flies were comparable to those in flies expressing hTau^2N4R^ alone (Fig. 5A,B). However, we observed an increase in the AT8 signal in flies co-expressing hTau^2N4R^ with MARK1, with or without TTBK2 (Fig. 5A,B).

**Fig. 6.**
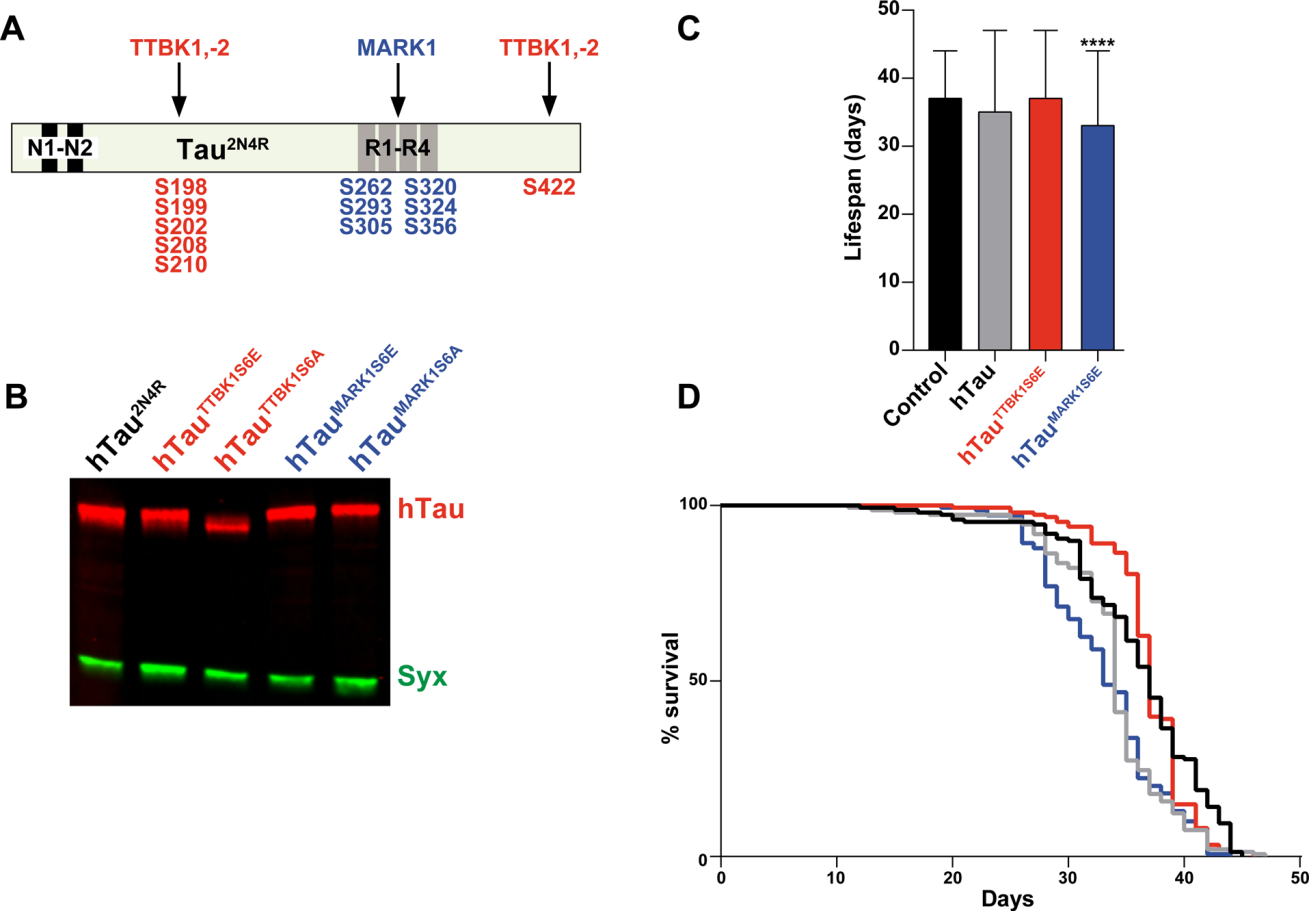
**Mutational analysis of phospho-residues in hTau.** (A) hTau^2N4R^ with the different phospho-serines that are known to be targeted by TTBK1, TTBK2 and MARK1. (B) Western blot analysis of mutant *UAS-hTau^2N4R^* transgenes driven by the *n-Syb-Gal4;Gal80[ts]* driver. hTau^2N4R^ was detected using a polyclonal hTau antibody; Syx was used as loading control (two independent experiments, 20 adult heads each). hTau^TTBK1S6A^ displayed faster migration than the other hTau^2N4R^ variants. (C,D) Lifespan analysis of mutant hTau^2N4R^ strains driven by the *n-Syb-Gal4;Gal80[ts]* driver. Results are depicted as Kaplan–Meier survival curves (D) (Kaplan and Meier, 1958) and bar graph (C). hTau^2N4R^ and hTau^TTBK1S6E^ did not show any toxicity while hTau^MARK1S6E^ toxicity was significant when compared to control flies (*n-Syb-Gal4;Gal80[ts]* crossed to the *attP* landing site strain BL#9750; error bars indicate mean±s.d.; ***P≤0.001; Dunnett's multiple comparison; *n*=3 independent experiments; ≥96 flies).

Next, we used a rabbit polyclonal antibody which detects phosphorylated S422, also known to be phosphorylated by TTBK1 and TTBK2 kinases (Fig. 6A). Again, we detected phosphorylation of hTau^2N4R^ at S422 in extracts from flies expressing hTau^2N4R^ alone, showing that hTau^2N4R^ is phosphorylated also at this site by endogenous *Drosophila* kinases (Fig. 5C). The phosphorylation of hTau at p422 by endogenous kinases has been previously observed (Grammenoudi et al., 2006). Looking at co-expressing flies, we observed an increase in the pS422 signal over total hTau^2N4R^ in flies co-expressing hTau^2N4R^ with TTBK1-MARK1 (Fig. 5C,D). In contrast, all other co-expression of hTau^2N4R^ with kinases did not result in elevated p422 level (Fig. 5C,D).

Together, these results indicate that while hTau^2N4R^ is already phosphorylated at several sites by endogenous kinases, this can be elevated by the co-expression of human TTBK1, TTBK2 or MARK1. Surprisingly, in spite of the co-toxicity of TTBK2 with hTau^2N4R^, as evidenced by lifespan and geotaxis, we did not observe any apparent effects on hTau^2N4R^ phosphorylation with TTBK2- hTau^2N4R^ co-misexpression.

### Phospho-residue mutagenesis supports a link between kinases and hTau toxicity

To further explore the nature of the hTau-kinase combinatorial toxicity, we next performed mutational analysis of hTau^2N4R^. To this end, we created mutant forms of hTau^2N4R^ that either prevents (S-to-A) or mimics (S-to-E) phosphorylation of the residues known to be phosphorylated by either TTBK1 (S198, S199, S202, S208, S210, S422) or MARK1 (S262, S293, S305, S320, S324, S356) (Fig. 6A) (Drewes et al., 1997; Sato et al., 2006; Timm et al., 2006; Chatterjee et al., 2009; Hanger et al., 2009; Lund et al., 2013; Ikezu and Ikezu, 2014). First, we analyzed the protein levels and mobility of these mutants by western blot (Fig. 6B). While the protein levels were comparable, we noticed that the hTau^2N4R^ mutant with S-to-A mutation in the TTBK1 sites (hTau^TTBK1S6A^) migrated faster than wild-type hTau^2N4R^ or hTau^MARK1S6A^ (Fig. 6B). This is in line with the positive signal for AT8 and pS422 already in flies expressing hTau^2N4R^ alone (Fig. 5). In contrast, we did not observe a clear mobility shift for the hTau^MARK1S6A^ mutant (Fig. 6B). Both of the phospho-mimic mutants (hTau^TTBK1S6E^ and hTau^MARK1S6E^) migrated with similar mobility as wild-type hTau^2N4R^ (Fig. 6B). To confirm the molecular behavior of these phospho-mutants, we analyzed western blots using phospho-antibodies. This revealed the anticipated loss of signal for the corresponding mutant-antibody pairs (Fig. S2A-C). In most cases the S6E mutants also resulted in loss of staining, presumably due to the difference in the structure of the p-Ser residue when compared to Glu (Fig. S2B,C). Noteworthy is that all phospho-antibodies tested reacted robustly with hTau^2N4R^ without co-expression of human kinases, demonstrating substantial phosphorylation of hTau^2N4R^ by endogenous *Drosophila* kinases (Fig. S2B,C).

Lifespan analysis, using the *n-Syb-Gal4;Gal80[ts]* driver, revealed that the hTau^TTBK1S6E^ phospho-mimetic mutant did not show toxicity when compared to wild-type hTau^2N4R^ or to control (*n-Syb-Gal4;Gal80[ts]* crossed to the *attP* landing site strain BL#9750) (Fig. 6C). However, in line with the fact that the MARK sites may not be highly phosphorylated by endogenous kinases, the hTau^MARK1S6E^ phospho-mimetic mutant showed more enhanced toxicity (Fig. 6C,D). To further address combinatorial toxicity between TTBK1 and hTau, we combined TTBK1 with an hTau mutant where the TTBK1 phospo-residues were mutated to alanine (hTau^TTBK1S6A^). This revealed that TTBK1 was still combinatorially toxic with hTau^TTBK1S6A^, when compared to TTBK1 alone (Fig. S3A,B).

## DISCUSSION

### Post-mitotic expression of hTau results in weak toxicity, and may more closely model the slow progression of tauopathies in post-mitotic neurons

A number of previous studies have relied on the *GMR-Gal4* and *elav-Gal4* drivers, both of which express in dividing cells: the developing eye disc and neural precursors, respectively (Freeman, 1996; Berger et al., 2007). Because of the microtubule-binding properties of Tau, its expression in mitotic cells may have toxic effects due to interference with mitotic spindles in dividing cells, and this notion is supported by recent studies (Bouge and Parmentier, 2016). In line with the potential of Tau toxicity in mitotic cells, expression of Tau, although widespread in the developing nervous system, is generally not observed in neural progenitors (Takemura et al., 1991). To circumvent the potentially non-physiological effects of Tau in dividing cells we have performed our toxicity studies of hTau in *Drosophila* using a post-mitotic pan-nervous system driver, the *n-Syb-Gal4* driver, driving throughout development and adulthood, or in an inducible version combined with *Gal80[ts]* to achieve selective expression only in adulthood. We also use the post-mitotic *OK371-Gal4* driver – a *Gal4 P* element enhancer trap insertion in the *Vesicular glutamate transporter* gene – expressed in cholinergic motor neurons, to address locomotor effects in a geotaxis assay. We would argue that the post-mitotic expression described here, achieved by using *n-Syb-Gal4* or *OK371-Gal4*, is more in line with the expression of Tau in post-mitotic cells, its function and dysfunction in neurons, as well as the generally slow progress of tauopathies in humans. Moreover, given the normally robust expression of Tau in most/all post-mitotic neurons throughout life, it is perhaps not surprising that we observe comparatively mild toxicity for hTau using post-mitotic drivers. Similar observations regarding the comparatively mild effects of hTau when expressed post-mitotically as opposed to mitotically has also been noted by others (Williams et al., 2000; Mudher et al., 2004; Folwell et al., 2010; Sinadinos et al., 2013).

### The six main isoforms of hTau show similar toxicity

Integrase-based site-specific transgenesis allowed us to address hTau isoform toxicity without the complicating issue of transgenic position effects upon expression levels. Other studies have provided support for the notion that certain isoforms are more toxic (van Slegtenhorst et al., 2000; Grammenoudi et al., 2008; Kosmidis et al., 2010). We did not find striking differences between isoforms when integrated at the same genomic position and driving them from the *n- Syb-Gal4* driver, although the hTau^2N3R^ isoform did show significantly higher toxicity when compared to hTau^2N4R^. This apparent lack of difference in toxicity for five of the isoforms and elevated toxicity for hTau^2N3R^ was not reflected by the protein analysis, where hTau^1N4R^ was the only isoform that showed significantly elevated protein levels, when compared to hTau^2N4R^. Combining the six hTau isoforms with TTBK1 we noted combinatorial toxicity, but we did not observe any striking differences in co-toxicity when comparing the six isoforms. We conclude that the six isoforms may well have differential toxicity in different settings, but would argue that these are rather minor differences.

### Combinatorial hTau-kinase expression results in increased toxicity

We find that post-mitotic expression of human TTBK1, TTBK2 and MARK1 kinases is toxic to the fly, even without co-expression of hTau. Comparing TTBK1 with TTBK2, we noted stronger effects on lifespan and geotaxis for TTBK1 than TTBK2, apparent in all three expression paradigms – direct *n-Syb-Gal4*, inducible *n-Syb-Gal4* (*n-Syb-Gal4;Gal80[ts]*) and *OK371-Gal4* (with or without hTau expression). Because both *UAS* transgenes were synthetic and contain identical 5′ and 3′ UTRs, are landed in the identical genomic location and the western blot show similar expression levels (Fig. 2B), we would speculate that these differences in toxicity truly reveal differences in the protein function of TTBK1 when compared to TTBK2. Intriguingly, TTBK1 and TTBK2 contain highly homologous kinase domains, but most of the sequences outside are not highly homologous (reviewed in Liao et al., 2015).

However, in spite of the toxicity observed when expressing these human kinases alone, we do observe combinatorial toxicity when they are co-expressed individually with hTau. This is apparent both in the lifespan and geotaxis assays. In addition, site-specific transgenesis allowed us to readily combine hTau with 2-3 kinases. This resulted in several interesting findings. First, regarding combinatorial kinase toxicity without hTau^2N4R^, we find that the combination of TTBK1 with MARK1 is highly combinatorially toxic. Specifically, while MARK1 alone is weakly toxic and TTBK1 reduces lifespan with 23%, their co-expression reduces lifespan by a non-additive 60% (Fig. 2C). Such combinatorial kinase toxicity may be due to action of common targets, on interacting targets, or on each other. Second, in contrast to combinatorial toxicity of TTBK1-MARK1, we did not find any enhancement of TTBK2 toxicity by co-expression of MARK1. Third, the TTBK1-TTBK2 double kinase flies show enhanced toxicity when compared to either kinase alone. Fourth, triple kinase flies are even more toxic, reduced down to 26% lifespan of control. These results point to a perhaps surprising selectivity in combinatorial kinase toxicity.

Next, focusing on the hTau-kinase combinations, we find that all hTau plus single kinase combinations were combinatorially more toxic than hTau or kinase alone. For double-kinase co-expression with hTau^2N4R^, the strongest effect was in the TTBK2-MARK1 combination, which was strongly enhanced by hTau^2N4R^ co-expression. In contrast, triple kinase expression, which again gave only a 26% lifespan when compared to control, was not significantly enhanced by hTau^2N4R^ co-expression (although there was a trend down). Surprisingly, TTBK1-TTBK2 co-expression, which is quite toxic, showed decreased toxicity when co-expressed with hTau^2N4R^.

We conclude that all three human kinases are toxic in flies, in a TTBK1>TTBK2>MARK1 order. Furthermore, all three are combinatorially toxic with hTau, and two of three double-kinase flies were also enhanced by hTau^2N4R^. However, triple kinase expression is highly toxic, and we can no longer observe any combinatorial toxic effect when co-expressing hTau^2N4R^. These results demonstrate that there is considerable toxicity of TTBK1, TTBK2 and MARK1 kinase expression even without hTau co-expression.

### Combinatorial hTau-kinase toxicity: direct or indirect action?

We observe combinatorial effects on toxicity when co-expressing TTBK1, TTBK2 or MARK1 kinase with hTau. A central question is whether or not this reflects a direct action of the kinases upon hTau, and hence increased hTau toxicity, or alternatively, totally independent toxicity mechanisms, which only combine with respect to shortening the lifespan or affecting geotaxis.

A number of studies have addressed hTau phospho-biology using *Drosophila* (Gistelinck et al., 2012; Fernandez-Funez et al., 2015; Sun and Chen, 2015). Several of these studies have provided support for the well-established notion that phosphorylation of specific hTau residues enhances toxicity. In addition, genetic screens in *Drosophila* and *C. elegans* have identified numerous kinases and phosphatases as suppressors/enhancers of Tau toxicity (reviewed in Hannan et al., 2016). Among the kinases identified were TTBK and MARK kinases (Shulman and Feany, 2003; Kraemer et al., 2006; Ambegaokar and Jackson, 2011).

Focusing on the MARK kinases, denoted Par-1 in *Drosophila*, previous studies have found that overexpression of Par-1 in the eye triggers a mild rough eye phenotype, which could be partly suppressed by removing one gene copy of dTau (Nishimura et al., 2004). Studies furthermore supported a role for the S262 and S356 residues, which are targeted by Par-1, and hTau toxicity (Nishimura et al., 2004; Chatterjee et al., 2009; Folwell et al., 2010; Iijima-Ando et al., 2010). We mutated the six main MARK kinase target sites in hTau, S-to-A or S-to-E, but did not observe any obvious mobility shifts in western blots for either mutant. However, we observed increased toxicity of the S6E mutant, when compared to hTau^2N4R^. Based upon these findings, we would postulate that hTau^2N4R^ is not extensively phosphorylated on the MARK sites by endogenous kinases, in spite of the presence of *Drosophila* Par-1. We further propose that MARK1 kinase over-expression increases toxicity, at least in part, via these sites. In relation to these observations it is noteworthy that the MARK1 sites on hTau are known to be involved in microtubule stabilization, i.e. when they are un-phosphorylated the microtubules are stabilized (Schneider et al., 1999; Matenia et al., 2005). Thus, this might be part of the explanation for MARK1-hTau combinatorial toxicity, especially in neurons given their reliance on fast axonal transport.

The TTBK kinases have been less studied with regard to hTau toxicity in *Drosophila*. However, studies have found enhanced toxicity linked to TTBK target sites in hTau. Single or double S-to-A mutations in two of the TTBK target sites (S199/S202) did not reduce toxicity (Steinhilb et al., 2007b). However, a 14 amino acid S/T-to-A mutant (including three TTBK sites: S199/S202/S422) did reduce hTau toxicity, and the converse S-to-E phospho-mimetic mutant increased toxicity (Steinhilb et al., 2007a). We find combinatorial toxicity between TTBK1 or TTBK2, when expressed with hTau. However, this combinatorial toxicity is still present in TTBK1 hTau^TTBK1S6A^ flies, and we did not observe enhanced toxicity in the phosho-mimetic hTau^TTBK1S6E^ mutant when expressed alone. Moreover, when co-expressing TTBK1, TTBK2 with hTau^2N4R^, we do not find a clear increase in pTau using AT8 (Fig. 5A,B). In contrast, TTBK1, but not TTBK2, resulted in an increase in pS422 (Fig. 5C,D). We observe faster migration of the S-to-A mutant on the western blots (Fig. 6B). Combined with the lack of enhanced toxicity in the S-to-E mutant, we propose that although pS422 can be enhanced, the TTBK target serines have a high degree of phosphorylation even without co-expression of TTBK1 or TTBK2 kinases, potentially by the *Drosophila* TTBK homolog Asator. Hence, in contrast to MARK1-hTau combinatorial toxicity, we propose that TTBK-hTau combinatorial toxicity may largely result from independent toxic effects of each protein.

One surprising finding was that while neither TTBK1 nor TTBK2 co-expression with hTau increased the AT8 signal, MARK1 triggered a minor increase (Fig. 5A,B). Because MARK1 is not known to target AT8 this is likely an indirect effect, either from triggering AT8 phosphorylation by acting on other ‘priming’ phospho-sites in hTau, or by triggering the activity of other kinases, e.g. TTBK1. It is tempting to speculate that such kinase cross-talk may help explain the strong co-toxicity observed when co-expressing MARK1 with TTBK1.

Recent studies have identified another kinase, Nuak1, which also phosphorylates Tau on S356 and hence shares a phospho-target site with MARK kinases (Lasagna-Reeves et al., 2016). However, unlike MARK kinases, which target several phospho-residues, Nuak1 exclusively targets S356. Nuak1 phosphorylation stabilizes Tau, and Nuak1 enhances Tau toxicity, both in flies and mice. It would be interesting to address combinatorial toxicity of MARK with Nuak1.

Finally, it should be noted that a growing number of studies are indicating that Tau phosphorylation may not be the only, or even primary, toxicity driver. For example, in a screen for modifiers of hTau toxicity, there was an apparent disconnect between modifier action and the extent of Tau phosphorylation, as well as the interaction of modifiers and wild type versus S11A phosphorylation-resistant mutant hTau (Ambegaokar and Jackson, 2011). In addition, zinc has been found to enhance Tau aggregation (Mo et al., 2009), and recent studies indicate that this operates independently of Tau phosphorylation status (Huang et al., 2014). Finally, recent studies of Tau toxicity have revealed a role for Tau toxicity and the loss of heterochromatin, indicating a nuclear role for Tau (Frost et al., 2014). Given the role of Tau in a number of human tauopathies, uncovering the function and dysfunction of Tau, its relationship to the growing list of Tau-kinases, and possible new roles for Tau, will continue to be important for future development of therapeutics.

## MATERIALS AND METHODS

### Fly stocks

From Bloomington Drosophila Stock Center: *UAS-hTau* (BL#51362) and *OK371-Gal4* (BL#26160). For this study, a double transgenic line, *n-Syb-Gal4#2-1, 1M* was used (Jonson et al., 2015). To generate *Gal80[ts]/n-Syb-Gal4*, because of the strong expression from the *n-Syb-Gal4* driver, two *tubP-Gal80[ts]* transgenes on chromosome 2 (BL#7019 and BL#7108) (McGuire et al., 2003) were recombined, and then combined with *n-Syb-Gal4#2-1, 1M*. This resulted in a driver line that showed no apparent eGFP expression in the adult fly brain when crossed to *UAS-eGFP* at 18°C, but robust expression when flies were moved to 29°C (Fig. S1).

### Generation of *UAS* Transgenic flies

Both hTau and kinases were codon optimized for expression in *Drosophila* (http://www.kazusa.or.jp/codon/). Sequences were added to the 5′: a consensus start codon (Cavener and Ray, 1991) and an EcoRI site, as well as to the 3′; three different stop codons (amb, och, opa) and an XbaI site (see Supplementary Materials for all DNA sequences). DNAs were generated by gene-synthesis (Genscript, New Jersey, USA), and cloned into pUAS.attB (Bischof et al., 2007), as EcoRI/XbaI fragments. DNAs were control-sequenced on both strands in the pUAS.attB vector (GATC BioTech, Germany), and injected into landing site strains BL#9723 (28E); BL#9736 (53B); BL#9750 (65B) and BL#9744 (89E) (BestGene, CA, USA).

### Lifespan assay

Lifespan assay using *n-Syb-Gal4* was performed as previously described (Jonson et al., 2015). Briefly, flies were reared at 25°C under a 12 h light:12 h dark cycle until eclosion and at 29°C post eclosion. Flies were maintained at 29°C in 50 ml vials (20 flies per vial) containing *Drosophila* food [water, potato mash powder, corn flour, yeast, agar, syrup, propionic acid (diluted: 48.5 ml propionic acid+∼950 ml H_2_O)]. Every 2-3 days the flies were transferred to fresh vials and the number of surviving flies was recorded throughout the lifetime of all flies. The assay was repeated three times and the data was pooled and analyzed together. For *n-Syb-Gal4;Gal80[ts]*, flies were kept at 20°C until eclosion, and flies were then reared at 29°C. As control, the different *Gal4* drivers used were crossed to the *attP* landing site strain BL#9750. GraphPad Prism 6.0a software (GrapPad Software Inc., San Diego, CA, USA) was used to generate Kaplan–Meier survival curves (Kaplan and Meier, 1958) and to perform all statistical analysis.

### Negative geotaxis assay

Transgenic UAS flies were crossed with *OK371-Gal4* line and reared at 26°C until eclosion. The adult flies were sorted and placed in 10 vials of 10 flies per vial and placed at 29°C. Only males were used in order to avoid differences in mobility due to amount of eggs carried by the fly. Flies were examined on Day 1, 7 and 14. Flies were always allowed to recover from CO_2_ for at least 3 h until assayed. Flies were transferred into new, empty vials and allowed to acclimatize for 30 s before the assay was started. The flies were gently shaken to the bottom of the vial and the percentage of flies that climbed up to a 5 cm mark on the vial within 30 s was counted, and the procedure was repeated 10 times for each vial. The mean with standard deviation is plotted.

### Western blotting of adult fly heads and antibodies

20 fly heads were homogenized in ice-cold RIPA buffer (10 mM Tris pH 8, 1 mM EDTA, 0.5 mM EGTA, 140 mM NaCl), 1 × protease inhibitors, 1 × PhosSTOP (both from Roche, Basel, Switzerland), and silica beads in a Fastprep24 homogenizer (MP Biomedical, Santa Ana, CA, USA) at 6.5* *m/s for 30 s, and quickly placed on ice. 2 × Laemmli protein sample buffer (BioRad) with 5% β-mercaptoethanol was added and the samples were then boiled for 5 min. The samples were then centrifuged at 12,000 ***g***, the supernatant transferred to a new tube and centrifuged again. SDS-PAGE, western blotting, blocking and antibody incubations were all performed according to standard procedures. Antibodies used were: mouse AT8 at 1:1000 dilution (Thermo Scientific), mouse Tau5 at 1:1000 dilution (Thermo Scientific), rabbit TauH-150 at 1:1000 dilution (Santa Cruz Biotechnology), mouse Syntaxin at 1:4000 dilution (mAb 8C3-s; Developmental Studies Hybridoma Bank), and mouse PHF1-Tau at 1:1000 dilution (kind gift from Peter Davies, Albert Einstein College of Medicine, Bronx, NY, USA). Rabbit monoclonal antibodies to pSTau198, pSTau199, pSTau202, pSTau262 and pSTau356, and rabbit polyclonal pSTau422, were all used at 1:1000 (Abcam). Secondary antibodies were all IRDye from Li-COR diluted 1:15,000: goat anti mouse 680RD, goat anti rabbit 680RD, goat anti rat 680RD, goat anti mouse 800CW, goat anti rabbit 800CW and goat anti rat 800CW. The nitrocellulose (Amersham Protran, GE Healthcare, Uppsala, Sweden) was blocked in 5% milk in PBS with 0.1% Tween for 30 min. Antibodies were diluted in 5% milk PBS with 0.1% tween.
